# African countries from the Pasteur Network reexamine their syndromic sentinel surveillance system associated with household contact within the AFROSCREEN program

**DOI:** 10.3389/fpubh.2023.1292435

**Published:** 2024-01-05

**Authors:** Mathurin Cyrille Tejiokem, Aliou Barry, Rila Ratovoson, Brice Yambiyo, Ramatoulaye Hamidou Lazoumar, Magali Herrant, Estelle Madaha, Vincent Richard

**Affiliations:** ^1^Centre Pasteur du Cameroun, Yaoundé, Cameroon; ^2^Institut Pasteur de Dakar, Dakar, Senegal; ^3^Institut Pasteur de Madagascar, Antananarivo, Madagascar; ^4^Institut Pasteur de Bangui, Bangui, Central African Republic; ^5^CERMES, Niamey, Niger; ^6^Institut Pasteur, Paris, France

**Keywords:** “pathogen X”, preparedness, epidemiology, sentinel surveillance system, syndromic surveillance, sequencing

## Abstract

Surveillance to better detect and respond to new pathogens remains a major challenge for global public health. The Pasteur Network recently held a brainstorming workshop located in Cameroon attended by Pasteur epidemiological teams from Niger, Central African Republic (CAR), Cameroon, Senegal, and Madagascar to discuss how the Pasteur Network in Africa could use the lessons of COVID-19 to set-up a pilot sentinel surveillance scheme given its expertise and involvement during the pandemic. The possibility of coupling sentinel syndromic and biological surveillance already implemented for influenza surveillance with the recent sequencing capacity put in place by the AFROSCREEN program prompted us to consider strengthening surveillance tools to target “Pathogen X” detection in Africa. The perspective project provided by the Pasteur Network teams and shared with other partners of the AFROSCREEN program will target strengthening of the diagnosis of severe acute respiratory infections (IRAS) and the surveillance of IRAS, the evaluation of the impact of SARS-CoV-2 on the epidemiology of IRAS, and the addition of the detection of new pathogens, called “Pathogen X,” based on sequencing capacity and epidemiological criteria from One Health approaches.

## Introduction

The rapid spread of respiratory viruses is a significant global concern because of pandemic risk. Recently, syndemic approaches have been stressed in response to this threat, focusing on inequity and social determinants (1, 2). Pandemic risk has long been considered for influenza viruses as their etiology is the best understood, in particular, that of avian influenza viruses. The recent global health crisis has shown that not only influenza viruses but also other respiratory viruses, such as the recent SARS-CoV-2, are high-risk pathogens that need to be considered in terms of surveillance. Keys to managing such risk are early detection and a rapid response. Relying on laboratory-confirmed cases for surveillance takes time, particularly in Africa, where laboratory capacity is limited. Therefore, the latest workshop, held in Cameroon in March 2023, brought together epidemiologists of the Pasteur Network involved in the AFROSCREEN program implemented by Centre Pasteur in Cameroon, Institut Pasteur de Dakar in Senegal, CERMES/Pasteur in Niger, Institut Pasteur d’Antananarivo in Madagascar, and Institut Pasteur de Bangui in the Central African Republic to brainstorm on future surveillance tools, taking into account the reduction of the financial support and the ongoing strengthening of laboratory capacity due to the AFROSCREEN program funded by the French Development Agency. This program has enabled the deployment of automated sequencers and has provided bioinformatics training related to the surveillance of SARS-CoV-2 variants.

Clearly, syndromic surveillance based on symptoms associated to syndromes (suspected cases) coupled with biological surveillance, based on laboratory diagnosis (confirmed cases), could be considered for first-line screening to circumvent long delays. By definition, syndromic surveillance is the act of collecting and analyzing patient symptom information before laboratory confirmation and identification of a signal corresponding to an outbreak or cluster is a main challenge.

However, with the strengthening of laboratory capacities, the future maintenance of the sequencing capacity implemented in response to the variants of SARS-CoV-2 and its use for surveillance will also be a critical issue.

The discussions at this meeting focused on how to maintain an acceptable and efficient monitoring capacity despite financial constraints.

## Subtopics

### History of sentinel surveillance of the Pasteur Network

The sentinel surveillance is usually conducted at specific sites for instance for influenza surveillance on voluntary bases for health stakeholders who report daily or weekly syndromic data. The first sentinel syndromic surveillance system set up within the Pasteur Network was implemented in Madagascar in 2007 using mobile phones and short message systems (3) as a means to collect and transfer daily data about fever syndromes from a limited number of primary healthcare centers designated as sentinel sites (4). It started with arbovirus surveillance related to the spread of Chikungunya in the Indian Ocean region, followed by influenza surveillance with nasal samples. This approach proved to be helpful for the detection of abnormal febrile events and diarrhea and tracking of the spread of the 2009 pandemic influenza virus (5). Although genuinely low cost, such surveillance necessitates the implementation of key elements and the collection of symptoms for evaluation purposes (6), raising the question of data quality and the sustainability of data collection, which are the cornerstones of syndromic approaches.

The second syndromic sentinel surveillance system based on febrile syndromes (influenza-like syndromes, dengue-like syndromes), was implemented in Senegal in 2012, based on the Madagascar model (7). The Senegalese sentinel syndromic surveillance network (“4S network”) is currently being used by health authorities and has enabled the detection of signals, leading to epidemiological investigations and confirmatory diagnoses by mobile laboratories and the eventual implementation of control measures.

These two Syndromic surveillance systems had been useful in detecting naturally occurring illness such as dengue, chikungunya, influenza A(H1N1)pdm09… (4, 5, 8).

### Lessons from the COVID-19 pandemic

**Figure 1:**
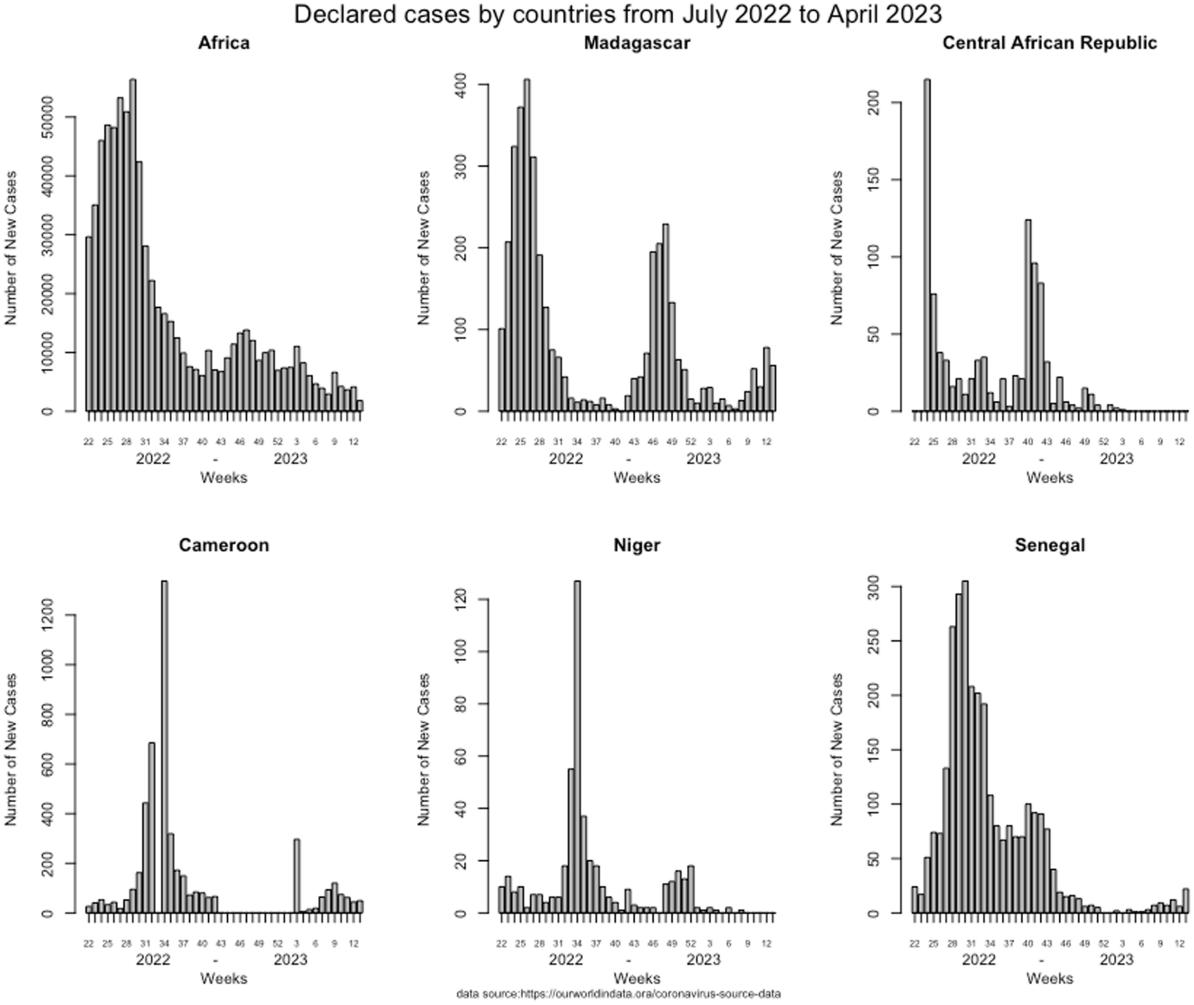
Weekly COVID-19 trends of declared cases from each of the five countries declared by the health authorities from July 2002 to April 2023.

**Figure 2:**
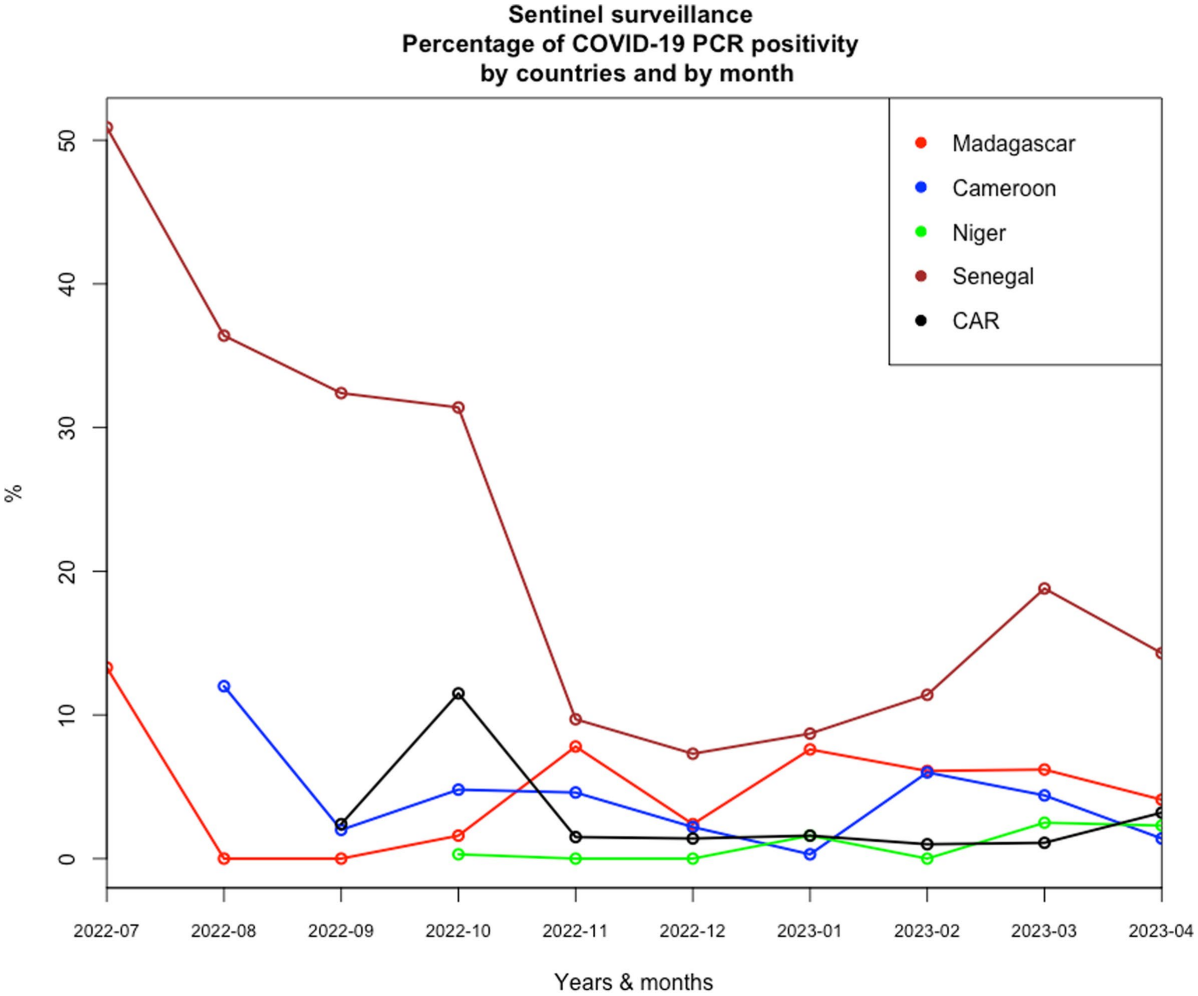
Monthly COVID-19 trends of positivity rates from sentinel surveillance in each of the five countries from July 2022 to April 2023.

Given the epidemiological situation of SARS-CoV-2 in Africa based on data declared internationally by health authorities of each country and collected on an opensource data collection,^1^ which has improved (Figure 1), with a decrease in all countries, we assessed the possibility of a syndromic sentinel surveillance approach (Figure 2) in CAR, Cameroon, Senegal, Madagascar, and Niger. This assessment was conducted in collaboration with epidemiologists, members of the Pasteur Network, and health authorities of each country based on the expertise and skills of Madagascar and Senegal and the momentum generated in the framework of the AFROSCREEN program, a consortium funded to promote SARS-CoV-2 sequencing in 13 African countries. The expected syndromic surveillance data was collected from primary healthcare centers and hospital emergency services and was able to detect new clusters of febrile and/or respiratory syndromes. Coupled with biological surveillance, syndromic surveillance detected the trends of the pandemic in each country. Figure 2 shows that the monthly COVID-19 trends from sentinel surveillance in each of the five countries are similar to the national reporting (Figure 1). This approach was further strengthened by the ability to detect high-risk variants due to the sequencing capacity of the program. Such capacity-building should be henceforth included in all surveillance designs and should be implemented for pathogen detection strategies.

How to be more reactive to detect new pathogens is a real challenge and need to develop experience sharing and collaboration for implementation of real-time like surveillance systems, syndromic surveillance coupled to laboratory-based surveillance In addition, the presence of dual epidemiological and biological skills on each Institut Pasteur in Africa and their historical collaborations with health authorities has been one of the keys to success and has made it possible to adapt the tools to the evolution of national policies, in particular with the contact tracing.

Key lessons for communicable disease surveillance in African countries arising from the COVID-19 pandemic are related to the promotion of national and regional collaborations and an interoperable surveillance system with the ability to detect new pathogens or new variants, monitor epidemic trends in real time, and assess the risks of spread in the population using relevant and measurable epidemiological indicators associated with public health epidemic intelligence.

## Discussion

This perspective piece is a reflection of a workshop hosted in Cameroon, attended by all authors, on March 2023 to consider how to maintain an acceptable and efficient monitoring capacity despite financial constraints.

In terms of real-time surveillance, sentinel syndromic surveillance using an electronic reporting platform, like the 4S network in Senegal (7), has become increasingly important given the risks of outbreaks related to febrile syndromes (malaria, arboviruses, acute respiratory infection) (8–10), even more so with the recent COVID-19 pandemic. Sentinel surveillance initially implemented for influenza surveillance helped track COVID-19 trends in each of the African countries included in the AFROSCREEN program. It would be highly useful to further develop such an approach, focusing also on interoperability in the framework of preparedness and response programs. Syndromic surveillance is one possible response to the lack of diagnostic capacity and the long turnaround times in low-income countries. It can be used not only for febrile syndromes, but also, for example, for diarrhea or dog bites for the assessment of rabies risk.

The real-time syndrome-based surveillance implemented in Madagascar and Senegal (3, 7) provides one of the quickest ways to identify multiple infectious diseases, for which fever is among the most common symptoms. The main goal of syndromic surveillance is to identify syndromic clusters early, before diagnoses are confirmed by laboratories and reported to public health authorities. Furthermore, the non-specific nature of syndromic surveillance and its timelier data reporting make it sufficiently sensitive and flexible to respond to a wide range of situations. In addition, developments in technology have facilitated improvements in data collection and accessibility of data from patient records.

Knowledge about the risk of emerging threats from a new pathogen is most often limited. Surveillance must therefore be associated with investigations of household contacts based, for example, on the free “Unit” protocols implemented by the WHO used in our five countries with limited results due to delay in implementation and the rapid fall of COVID-19 cases since September 2022 (Figure 2). Lessons learned from the COVID-19 study should be used to establish new operational surveillance tools associated with the investigation of household contact to increase the chances of finding new pathogens or variants and collecting epidemiological data on their transmission at the community level.

As international travel revitalizes and favors multiple types of exchanges, collaborative programs are essential, because infectious diseases will clearly remain a serious concern, with the need for their control across regional borders. At the stage of preparedness, collaboration is critical, and the implementation and evaluation of accurate surveillance systems are crucial for preventing the global spread of infectious diseases.

Collaborations between epidemiologist and biologist, like what is done in the Pasteur institutes, at the national, regional, and international level are clearly the best way to direct appropriate funding for the rapid adaptation of response measures to any outbreak scenario and to help identify specific etiologies. This approach would be aided by strengthening of the Pasteur Network, its broad international scope, and its capacity to implement collaborations at various levels: local, regional, and international. Such collaborations would involve public health authorities to coordinate the stakeholders and ensure the sustainability of national sentinel surveillance networks that combine both epidemiological and biological surveillance, including sequencing. There is a true potential for further development of the system, collaborative work, and harmonization between countries.

However, the question of the sustainability of financial support that had emerged during the meeting in Cameroon has led to propose a smaller model focused on severe acute respiratory infections, a target to be preferred for the identification of new pathogens or variants.

## Conclusion

The Pasteur Network teams must take advantage of the recent lessons learned from the COVID-19 pandemic with the deployment of new and cutting-edge diagnostic resources in developing future surveillance programs combining epidemiological, biological surveillance (+ sequencing), and contact tracing focused on the detection of “Pathogen X” based on SARI surveillance. Such efforts will require the sharing of any acquired skills with national, regional, and international organizations and institutions in charge of global public health decision making. Furthermore, these collaborations will have to conduct or adapt the preventive programs implemented by health authorities.

## Data availability statement

The original contributions presented in the study are included in the article/supplementary material, further inquiries can be directed to the corresponding author.

## Author contributions

MT: Writing – review & editing, Investigation. AB: Writing – review & editing, Investigation. RR: Writing – review & editing, Investigation. BY: Writing – review & editing, Investigation. RH: Writing – review & editing, Investigation. MH: Writing – review & editing, Project administration. EM: Writing – review & editing, Project administration. VR: Project administration, Writing – original draft, Writing – review & editing.

## Footnotes

^1^ https://ourworldindata.ora/coronavirus-source-data
